# Ultrasonic Cavity Preparation with CVD Diamond Tips: A Scanning Electron Microscopy Study of Dentin Surface Morphology and Smear Layer Formation

**DOI:** 10.4317/jced.64155

**Published:** 2026-05-29

**Authors:** Mauricio Schneider, Jefferson David Melo de Matos, Mário Alexandre Coelho Sinhoreti, Diego Telomaro Peiter, José Eduardo Bastian, Daher Antonio Queiroz

**Affiliations:** 1 DDS, MS, Assistant Professor, Department of Prosthodontics, Nova Southeastern University College of Dental Medicine, Fort Lauderdale, Florida, USA; 2 PhD, Department of Biomaterials, Dental Materials and Prosthodontics, São Paulo State University (Unesp), Institute of Science and Technology, São José dos Campos, Sao Paulo, Brazil; 3 Professor, Department of Restorative Dentistry, Dental Materials Division, Piracicaba Dental School (FOP UNICAMP), Piracicaba, Sao Paulo, Brazil; 4 DDS, Department of Dentistry Universidade Luterana do Brasil (ULBRA), Torres, Rio Grande do Sul, Brazil; 5 DDS, MS, PhD, Associate Professor, Department of Restorative Sciences and Public Health Dentistry, Nova Southeastern University College of Dental Medicine, Fort Lauderdale, Florida, USA; 6 Adjunct Associate Professor, Department of Restorative Dentistry &amp; Prosthodontics, UTHealth School of Dentistry, Houston, Texas, USA

## Abstract

**Background:**

Minimally invasive dentistry has driven the development of alternative cavity preparation techniques aimed at preserving tooth structure and improving biological outcomes. Ultrasonic systems associated with chemical vapor deposition (CVD) diamond tips have emerged as a potential alternative to conventional rotary instruments; however, evidence regarding their effects on dentin surface morphology and smear layer characteristics under SEM remains limited. This study aimed to evaluate the morphological characteristics of dentin surfaces after cavity preparation using ultrasonic and conventional rotary techniques.

**Materials and Methods:**

Twenty extracted human teeth were sectioned longitudinally to obtain standardized specimens. Samples were assigned to ultrasonic preparation using CVD diamond tips or conventional rotary preparation using carbide burs at high and low speeds. After preparation, specimens were cleaned, stored in distilled water, sputter-coated, and analyzed by scanning electron microscopy (SEM). Surface morphology, smear layer presence, and dentinal tubule exposure were qualitatively assessed at different magnifications.

**Results:**

Ultrasonic preparation resulted in consistently cleaner dentin surfaces with minimal smear layer and open dentinal tubules, whereas conventional rotary instrumentation produced irregular surfaces covered by dense smear layer and debris.

**Conclusions:**

Ultrasonic cavity preparation using CVD diamond tips demonstrated superior surface characteristics compared with conventional rotary techniques, supporting its potential as a minimally invasive approach that may enhance adhesion and preserve dentin-pulp integrity.

## Introduction

The evolution of restorative dentistry has progressively shifted from mechanically driven, extension-based cavity designs toward biologically oriented, minimally invasive approaches (1 , 2). Traditionally, cavity preparation has relied on high-speed rotary instruments equipped with electroplated diamond burs, which remain widely used due to their efficiency in removing dental hard tissues (3 , 4). However, these instruments present inherent limitations, including restricted visibility of the operative field, reduced tactile sensitivity, and the potential for unnecessary removal of sound tooth structure (5). Contemporary restorative strategies emphasize the preservation of healthy and remineralizable tissues, in line with the principles of minimally invasive dentistry (6 , 7). This paradigm shift has led to the development and clinical adoption of alternative technologies aimed at enhancing precision, selectivity, and patient comfort during cavity preparation (8). Among these, oscillating instruments, particularly ultrasonic systems, have attracted increasing interest due to their conservative cutting mechanisms and improved operator control (9 , 10). Oscillating instruments, classified as sonic or ultrasonic according to their frequency range, were first introduced into dentistry in the mid-20th century (11). Although their early clinical application was limited by reduced cutting efficiency and restricted tip availability, subsequent technological advancements have significantly expanded their capabilities and clinical indications (12 , 13). Modern ultrasonic systems incorporate optimized tip geometries and advanced diamond coatings, enabling improved performance across a range of procedures, including restorative, periodontal, and endodontic treatments (14). Compared with conventional rotary systems, ultrasonic instruments offer several potential advantages. Their smaller working tips enhance visualization of the operative field, while their oscillatory motion allows for more selective removal of dental tissues (15). In addition, these systems are associated with reduced noise, vibration, and patient discomfort, which may improve patient acceptance, particularly in pediatric and anxious individuals (16 - 18). Nevertheless, concerns persist regarding their lower efficiency in enamel removal, which continues to limit their widespread use in routine cavity preparation (13). The introduction of chemical vapor deposition (CVD) diamond technology represents a further advancement in ultrasonic instrumentation. Unlike conventional diamond burs, which rely on mechanically bonded abrasive particles, CVD-coated tips provide a continuous, highly adherent diamond layer, resulting in improved durability, cutting efficiency, and reduced particle detachment (19 , 20). These characteristics may enhance the clinical performance of ultrasonic systems, particularly with respect to precision, surface quality, and biological compatibility (21). From a biological standpoint, cavity preparation inevitably induces mechanical and thermal stresses on the dentin-pulp complex (22). Excessive heat generation, especially in the absence of adequate cooling, may result in irreversible pulpal damage (23). Moreover, the characteristics of the prepared dentin surface, including smear layer formation and dentinal tubule occlusion, play a critical role in influencing adhesion, permeability, and overall restorative success (24 - 26). Therefore, the selection of appropriate instrumentation and technique is essential for preserving tissue integrity and optimizing clinical outcomes (27). Given these considerations, the investigation of alternative preparation methods that combine efficiency, precision, and biological safety remains highly relevant. Therefore, the aim of this study was to evaluate, by means of scanning electron microscopy (SEM), the effects of dentin cavity preparation using two different techniques: conventional high-speed rotary instrumentation and ultrasonic preparation with CVD diamond tips, focusing on surface morphology, structural integrity, and potential biological implications.

## Materials and Methods

1. Sample Selection Twenty extracted human teeth were obtained from the Human Tooth Bank (Biobank) of the Universidade Luterana do Brasil, Campus Torres, RS, Brazil (protocol B-090). All procedures complied with institutional ethical guidelines for the use of human dental tissues. Each tooth was sectioned longitudinally to obtain standardized specimens. Representative specimens from each group were selected and labeled (A-D) for qualitative SEM analysis. 2. Specimen preparation All teeth were sectioned using a low-speed carborundum disc (KaVo®) under continuous water cooling to prevent thermal damage. Following sectioning, the specimens were sequentially polished using silicon carbide abrasive papers (100-, 400-, and 600-grit) under water irrigation to obtain flat, standardized, and homogeneous surfaces. 3. Experimental Design The specimens were divided into two experimental groups according to the preparation technique employed: - Group I (Tooth I) Sample A (Ultrasonic preparation): A standardized Class I cavity was prepared using a Gnatus® ultrasonic device with CVDentus® diamond tips. Sample B (Conventional rotary preparation): A Class I cavity was prepared using a high-speed #245 carbide bur (S.S. White Dental Inc.®) under water spray for enamel removal. Dentin was subsequently prepared using a low-speed #6 round bur. - Group II (Tooth II) Sample C (Ultrasonic preparation): The entire specimen surface was prepared using a Gnatus® ultrasonic device with CVDentus® diamond tips. Sample D (Conventional rotary preparation): The enamel surface was prepared using a high-speed #245 carbide bur under water cooling, followed by dentin preparation with a low-speed #6 round bur. 4. Post-Preparation Procedures Following cavity preparation, all specimens were rinsed with an air-water spray to remove debris and stored in distilled water to maintain hydration until analysis. Subsequently, the samples were dehydrated, sputter-coated with a conductive metal layer, and examined using scanning electron microscopy (SEM; Philips XL 20) at the Universidade Luterana do Brasil, Canoas Campus. 5. Statistical analysis Due to the qualitative nature of the analysis, no statistical tests were performed. The evaluation was descriptive, based on SEM observations at different magnifications.

## Results

**Figure 1:**
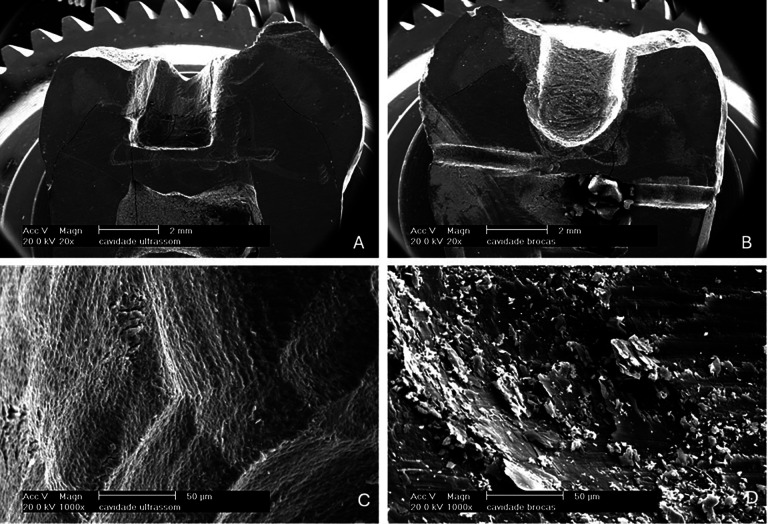
Scanning electron micrographs of dentin surfaces after cavity preparation. (A) Ultrasonic preparation (Sample A, 20×) showing a well-defined cavity outline and smooth surface. (B) Conventional rotary preparation (Sample B, 20×) demonstrating a less regular surface with reduced definition. (C) Ultrasonic preparation (Sample A, 1000×) revealing open dentinal tubules and minimal smear layer. (D) Conventional rotary preparation (Sample B, 1000×) showing smear layer and surface debris.

All specimens were successfully obtained and analyzed at the predetermined time intervals, with no sample loss during the experimental period. 1. Tooth I (Samples A and B) At low magnification (20×), the ultrasonic preparation (Sample A) exhibited a more clearly defined cavity outline with smoother surface morphology (Fig. 1A), whereas the conventional rotary preparation (Sample B) demonstrated a less regular surface with reduced definition (Fig. 1B).

1

**Figure 2:**
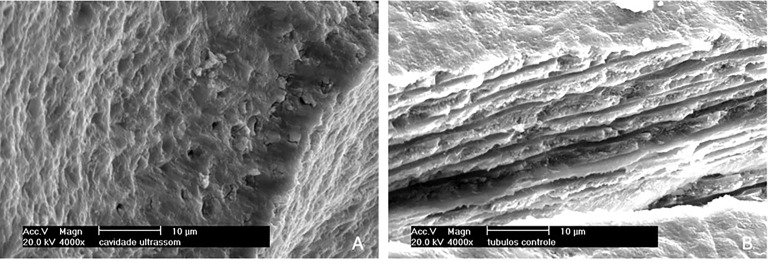
Scanning electron micrographs (4000×) of dentin surfaces after cavity preparation. (A) Ultrasonic preparation (Sample A) showing clearly visible dentinal tubule openings and minimal smear layer. (B) Conventional rotary preparation (Sample B) revealing a dentinal crack, likely associated with dehydration during specimen preparation, and the orientation of dentinal tubules.

At higher magnification (1000×), Sample A (Fig. 1C) revealed a porous dentin surface with open tubules, consistent with minimal or absent smear layer. In contrast, Sample B (Fig. 1D) showed a surface covered by debris, indicative of smear layer formation. At 4000× magnification, Sample A (Fig. 2A) demonstrated clearly visible dentinal tubule openings and an almost complete absence of smear layer.

2

**Figure 3:**
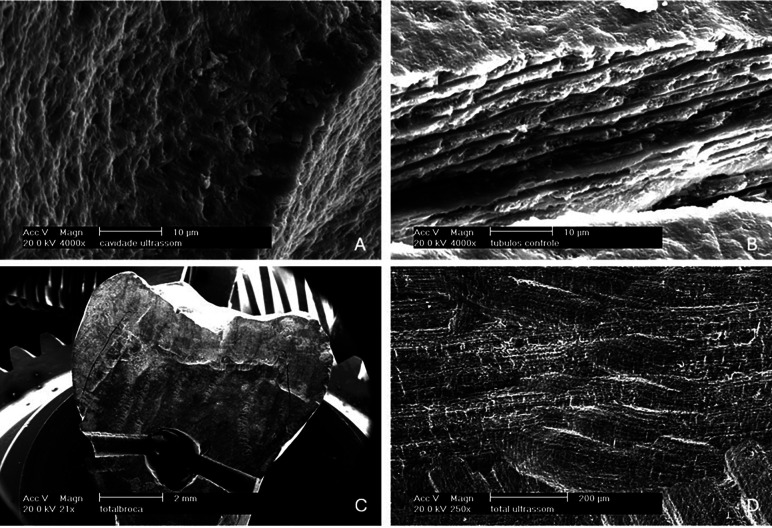
Scanning electron micrographs of dentin surfaces. (A) Standardized polished surface prior to cavity preparation, representing the baseline condition. (B) Ultrasonic preparation (Sample C) showing a clean and homogeneous surface. (C) Conventional rotary preparation (Sample D) revealing residual debris and irregular morphology. (D) Ultrasonic preparation (Sample C, 250×) demonstrating a homogeneous surface with grooves produced by the CVD diamond tip.

In Sample B (Fig. 2B), a dentinal crack was observed, likely resulting from dehydration during specimen preparation, highlighting the orientation of dentinal tubules. 2. Tooth II (Samples C and D) Figure 3 illustrates the standardized, polished surface to which all specimens were subjected prior to cavity preparation. Comparative analysis between Samples C and D (Figs. 3A,B,C) revealed a markedly cleaner dentin surface following ultrasonic preparation. In contrast, the surface prepared with the low-speed round bur exhibited residual debris and a more irregular morphology.

3

**Figure 4:**
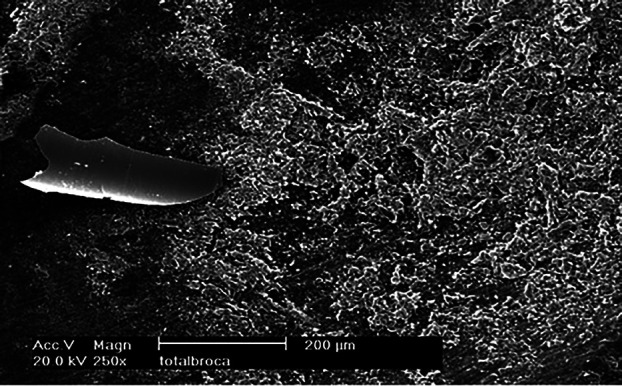
Scanning electron micrograph (250×) of dentin surface after conventional rotary preparation (Sample D), showing a dense smear layer with embedded debris and particles consistent with bur wear.

At 250× magnification, Sample C (Fig. 3D) displayed a homogeneous and clean surface, with visible grooves corresponding to the action of the CVDentus® tip. Conversely, Sample D (Fig. 4) exhibited a dense smear layer ("dentin mud"), including embedded debris.

4

Notably, a fragment consistent with bur wear debris was observed within the smear layer, suggesting possible instrument wear during preparation.

## Discussion

The present study evaluated the morphological characteristics of dentin surfaces following cavity preparation using ultrasonic instrumentation with CVD diamond tips compared with conventional rotary systems. The findings demonstrated that ultrasonic preparation resulted in cleaner dentin surfaces, greater preservation of dentinal microstructure, and reduced smear layer formation when compared with conventional techniques. At low magnification, ultrasonic preparations exhibited more defined and regular cavity margins. This may be attributed to the controlled oscillatory motion and reduced mechanical aggressiveness of ultrasonic systems, which allow more precise and conservative tissue removal. These findings are consistent with previous studies reporting improved marginal integrity and enhanced control associated with ultrasonic instrumentation, particularly in minimally invasive procedures (1 , 2 , 9). At higher magnifications, the most relevant difference between the two techniques was related to the condition of the dentin surface. Specimens prepared with ultrasonic tips showed open dentinal tubules and minimal smear layer formation, whereas those treated with rotary instruments exhibited a dense smear layer covering the surface. This observation is in agreement with previous reports demonstrating that sono-abrasion techniques produce significantly less smear layer compared with conventional rotary preparation (8 , 21 , 22). From a clinical standpoint, this difference is highly relevant, as the smear layer plays a critical role in adhesive performance. Although the smear layer may act as a temporary barrier by reducing dentin permeability, it can also hinder adhesive penetration and compromise bonding effectiveness (5 - 7). Therefore, the reduced smear layer observed in ultrasonic preparations may represent a significant advantage, as cleaner dentin surfaces with open tubules are more favorable for resin infiltration and hybrid layer formation. Consequently, ultrasonic preparation may contribute to improved bonding quality and long-term restoration durability. Another important finding of this study was the absence of debris and the overall cleaner surface observed in specimens prepared with CVD ultrasonic tips. In contrast, rotary instrumentation resulted in the accumulation of dentin debris and the presence of foreign particles embedded within the smear layer. This is likely associated with the abrasive and frictional cutting mechanism of rotary instruments, which generates more debris and frictional heat. Previous studies have also reported that conventional burs may undergo wear and release particles during use, potentially contaminating the prepared surface (8). The improved surface characteristics observed with ultrasonic preparation may also have biological implications. Reduced smear layer formation, together with lower thermal and mechanical stress, may contribute to better preservation of the dentin-pulp complex. In this context, previous investigations have demonstrated that ultrasonic systems are associated with lower temperature increases compared to high-speed rotary instruments, particularly when adequate cooling is employed (9). Additionally, more favorable pulpal responses, including reduced inflammatory reactions, have been reported following ultrasonic preparation (10). From a clinical perspective, ultrasonic systems offer additional advantages, including improved visibility, reduced noise, and lower vibration levels. These factors may enhance patient comfort and acceptance, particularly in pediatric and anxious individuals (11 - 13). However, despite these benefits, the lower cutting efficiency of ultrasonic instruments, especially in enamel, remains a limitation, as highlighted in previous studies (14). Therefore, a combined approach integrating both rotary and ultrasonic systems may represent a clinically effective strategy in specific situations. These findings further support the clinical relevance of ultrasonic systems in minimally invasive restorative dentistry. Some limitations of the present study should be acknowledged. The relatively limited sample size and the in vitro design may restrict the generalizability of the findings. In addition, artifacts such as dentinal cracks observed in some specimens may be associated with the dehydration process required for SEM analysis. Future studies incorporating larger sample sizes, quantitative methodologies, and in vivo conditions are recommended to further validate these results and explore their clinical implications.

## Conclusions

Ultrasonic cavity preparation using CVD diamond tips demonstrated superior dentin surface characteristics compared with conventional rotary instrumentation, supporting its potential as a minimally invasive alternative capable of enhancing adhesive performance and preserving dentin-pulp integrity.

## Data Availability

The datasets generated and analyzed during the current study are available from the corresponding author upon reasonable request.
